# Tuberculosis vaccine developments and efficient delivery systems: A comprehensive appraisal

**DOI:** 10.1016/j.heliyon.2024.e26193

**Published:** 2024-02-14

**Authors:** Rasoul Hoseinpour, Alka Hasani, Behzad Baradaran, Jalal Abdolalizadeh, Roya Salehi, Akbar Hasani, Edris Nabizadeh, Mina Yekani, Roqaiyeh Hasani, Hossein Samadi Kafil, Khalil Azizian, Mohammad Yousef Memar

**Affiliations:** a Infectious and Tropical Diseases Research Center, Tabriz University of Medical Sciences, Tabriz, Iran; b Immunology Research Center (IRC), Tabriz University of Medical Sciences, Tabriz, Iran; c Department of Laboratory sciences and Microbiology, Faculty of Medicine, Tabriz Medical Sciences, Islamic Azad University, Tabriz, Iran; d Department of Medical Microbiology, Faculty of Medicine, Tabriz University of Medical Sciences, Tabriz, Iran; e Clinical Research Development Unit, Sina Educational, Research, and Treatment Center, Faculty of Medicine, Tabriz University of Medical Sciences, Tabriz, Iran; f Drug Applied Research Center and Department of Medical Nanotechnology, Faculty of Advanced Medical Sciences, Tabriz University of Medical Sciences, Tabriz, Iran; g Department of Clinical Biochemistry and Applied Sciences, Tabriz University of Medical Sciences, Tabriz, Iran; h Department of Microbiology, Faculty of Medicine, Kashan University of Medical Sciences, Kashan, Iran; i Student Research Committee, Kashan University of Medical Sciences, Kashan, Iran; j Istanbul Okan University, 34959 Tuzla/İstanbul, Turkey; k Department of Microbiology, Faculty of Medicine, Kurdistan University of Medical Science, Sanandaj, Iran

**Keywords:** Tuberculosis, Vaccine, Delivery systems, Bacillus Calmette–Guérin

## Abstract

Despite the widespread use of the Bacillus Calmette–Guérin (BCG) vaccine, *Mycobacterium tuberculosis* (MTB) continues to be a global burden. Vaccination has been proposed to prevent and treat tuberculosis (TB) infection, and several of them are in different phases of clinical trials. Though vaccine production is in progress but requires more attention. There are several TB vaccines in the trial phase, most of which are based on a combination of proteins/adjuvants or recombinant viral vectors used for selected MTB antigens. In this review, we attempted to discuss different types of TB vaccines based on the vaccine composition, the immune responses generated, and their clinical trial phases. Furthermore, we have briefly overviewed the effective delivery systems used for the TB vaccine and their effectiveness in different vaccines.

## 1 Introduction

**Fig. 1:**
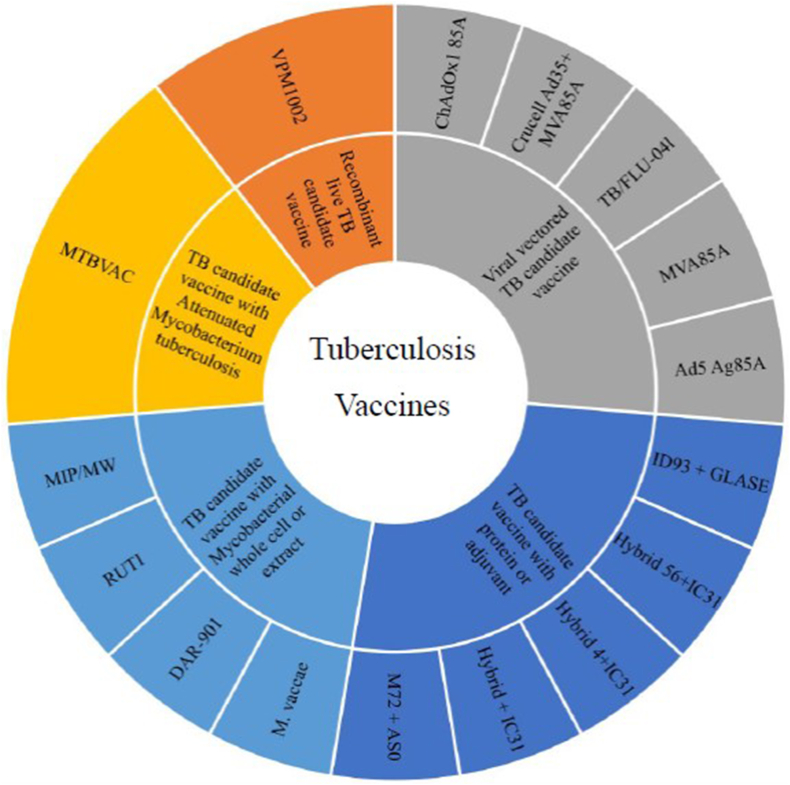
Types of tuberculosis vaccines based on composition.

New introduced TB (Tuberculosis) vaccines, show effective potential to eradicate of *Mycobacterium Tuberculosis* (MTB) and to induce immune response thereby preventing further new infections [1,2]. The Global Tuberculosis Report 2022 was issued by the World Health Organization on October 27. This report provides a comprehensive evaluation of the worldwide TB situation, drawing on data gathered from 202 countries and territories. The information covers over 99% of the global population and TB cases. Approximately 10.6 million individuals contracted TB in 2021, marking an increase from the 10.1 million cases documented in 2020. The death toll from TB in 2021 reached 1.6 million, including 187,000 patients with HIV, compared to 1.5 million in 2020, which included 214,000 patients with HIV [3]. The risk of TB recurrence from latency, which can arise years after primary infection, can be avoided by these types of vaccines with antigens expressed via MTB bacteria during latent-phase infection [4,5]. The findings of some studies have raised hopes for the establishment and development of TB vaccines even more effective than Bacillus Calmette–Guérin (BCG). Besides, more effective control of TB requires increased public awareness, political, funding, and international support [1]. In addition, several novel strategies are currently being studied to improve of TB vaccines. BCG vaccine was made by attenuating the *Mycobacterium bovis* strain against TB by Albert Calmette and Camille Guerin in 1921 [6]. BCG vaccination is commonly used in TB-endemic countries because it provides effective protection against the infant type of TB. In these areas, newborns are vaccinated with a single intradermal dose as early as possible soon after birth [[7], [8], [9]]. Today, globally about 120 million doses of BCG vaccine are administered to infants every year [2]. BCG vaccine may induce some side effects, but they're uncommon and generally mild. More serious complications, such as abscesses, bone inflammation, and widespread TB are very rare. BCG vaccine is 70–80% effective against the most severe forms of TB, such as TB meningitis. However, it is less effective in preventing pulmonary TB and despite its proven efficacy, protection is still questionable in immunocompromised or immunocompetent individuals. While the BCG vaccine demonstrates over 80 percent effectiveness in conferring resistance against active TB in children, its protective impact is inadequate for adults. Due to its unsatisfactory ability to prevent pulmonary TB in adults, the BCG vaccine has not succeeded in significantly reducing the global burden of TB. Another limitation lies in its impact on the tuberculin skin test, making it impractical for individuals with compromised cellular immunity, and its failure to induce cytotoxic CD8⁺ responses pose additional challenges. Consequently, there is a need to implement alternative strategies for developing a new TB vaccine. Two broad approaches can be considered: firstly, replacing BCG with other vaccines that enhance cellular immunity, and secondly, employing distinct vaccines that serve as boosters to reinforce the effectiveness of BCG [[10], [11], [12]]. Currently, the main focus of most developing TB vaccines is based on the production of new vaccines instead of BCG or using them as the booster of BCG [13]. There are various TB vaccines in the trial phase which includes three groups: therapeutic vaccines, priming, and prime boosters vaccines [14]. Most of these vaccines are based on a combination of proteins/adjuvants or recombinant viral vectors used for certain MTB antigens [15]. Knowledge the status of existing and developing options for TB vaccine as well as vaccine delivery systems allows researchers in this field to more effectively identify new vaccines and their limitation. In this review, we attempted to present some of the new findings about the types of TB vaccine candidates and their performance on the immune system and their clinical trial stages (Figure-1) along with the delivery systems. For this purpose, data on TB vaccines and delivery system of TB vaccines were detected in databases of PubMed, Scopus and the Google Scholar. The internet searches were done to find published articles with the keyword's MTB, TB, vaccine, delivery systems. All English language articles were found and read independently by two individuals.

## 2 TB candidate vaccine with protein or adjuvant

### 2.1 M72 + AS0

A recombinant vaccination called M72/AS01E was introduced to enhance the immunological response induced by BCG or MTB. The M72 antigen and Mtb72F, a fusion protein made up of the Mtb32a and Mtb39a antigens, have a close relationship. To increase the long-term sustainability of Mtb72F, a point mutation was made in the Mtb32a antigen of M72. The Quillaja saponaria fraction 1 (QS21) and immunostimulants monophosphoryl lipid (MPL) are mixed with adjuvant in the liposomes system known as AS01E. It triggers TH1 cellular and humoral responses [16,17].

In both cases of MTB-infected and uninfected healthy individuals from a TB endemic area, M72/AS01E vaccination has been shown to generate an acceptable immunity and reactogenicity profile and produced potent and sustained CD4 and CD8 T cell responses, as well as CD4⁺ T cell dependent IFN- recall responses in NK cells [18], and two M72/AS01 doses produced higher immune responses than a single dose [19].

Phase 2b controlled trial revealed that 54.0% protection was provided by M72/AS01E for MTB*–*infected adults, without evident safety concerns. Additionally, cases of pulmonary TB among healthy MTB-infected, BCG-vaccinated, and HIV-negative adults were significantly lower with M72/AS01E than with placebo [20], another study revealed approximately 50% protection against progression to active pulmonary TB for 3 years in MTB-infected, HIV-negative adults [21], and there was significant decrease in bacteriologically confirmed pulmonary TB [22].

Kumarasamy et al. [23], reported that M72/AS01E vaccine induced 3-year humoral and cellular immune responses in both HIV- and HIV + adults without safety concerns. Therefore, M72/AS01E is the first TB vaccine against TB in a century that had significant efficacy [22,24,25].

### 2.2 Hybrid + IC31

Low efficacy and risk of disseminated BCG especially in immunocompromised individuals motivated to use a vaccine that can provide protective immunity response to TB by inducing Th1-type cellular immune response characterized by interferon-gamma (IFN-γ) production. Hybrid 1-IC31, which combines IC31 with ESAT-6 and antigen 85B (Ag85B), is utilized as an adjuvant. This vaccine comprises of H1 which is made up of the two secreted antigens Ag85B and ESAT6. IC31 that can stimulate robust IFN-γ production by CD4 and Th1 cells in humans and maintain long-lasting memory immune responses has been tested in combination with H1. In phase I clinical trial, H1/IC31 has been proved to be safe in healthy adults with no adverse events reported and can induce strong and lasting Th1 responses. Despite MTB infection, it primes long-lived CD4⁺ T cell responses that cause children to produce TNF-α and IL-2 [26]. Two injections of the 15g of H1:IC31 dosage has been shown to be effective for the strongest immunological response (South African National Clinical Trials Register, DoH-27-0612-3947; Pan African Clinical Trial Registry, PACTR201403000464306). Injection of H1 with IC31 adjuvant intramuscularly has been shown by Van Dissel et al. [27] to be safe and well-tolerated in mycobacterially uninitiated individuals, and vaccination led to specific, potent, and long-lasting Th1-type immune responsiveness to the vaccine antigens. A robust cellular immunological response has also been reported to be promoted by a single dose of antigen adjuvanted with IC31®, which was further enhanced by booster immunization. Consequently, two H1:IC31 vaccinations at the 15g dose may be an effective TB vaccine candidate for adolescents [[27], [28], [29]]. The results of Phase I trial on H1/IC31® in individuals living in TB-endemic areas in a revealed that this vaccine was safe and mostly well-tolerated, especially in TB endemic areas [30]. H1/IC31 was also well-tolerated and safe in HIV-infected people with a CD4⁺ lymphocyte count greater than 350 cells/mm. HIV-1 viral load or the number of CD4⁺ T cells were unaffected by this vaccination. Vaccination with H1/IC31 elicited a potent local Th1 immune response [31].

### 2.3 Hybrid 4 + IC31

The Statens Serum Institut (Copenhagen, Denmark) developed HyVac4 (H4), which combines the adjuvant IC31 with the antigens Ag85B and TB10.4. One of the most crucial ESAT-6-like proteins is TB10.4. TB10.4 induced the production of TB10.4-specific IL-2 or TNF-α, CD4⁺ T cells, TNF-α and IFN-γ in BCG-vaccinated mice [32]. After being used in a prime/boost regimen, H4 expressed in IC31 provided superior protection against TB in the guinea pig model than the BCG vaccination alone [33]. H4:IC31 triggered a long-lasting Th1 response in a phase I trial study in uninfected, BCG-vaccinated individuals and had an acceptable safety feature. Higher CD4⁺ T cell response with functional traits that have been shown to be protective in vaccinated animal models by the 15 g dosage [34]. A Phase II clinical trial, supported by 10.13039/100014588Sanofi Pasteur, 10.13039/100009342Aeras, and the HIV Vaccine Trials Network, is presently started to assess the safety and immunogenicity of the vaccine in infants who have received BCG vaccination. Currently, phase II studies are underway in Africa to assess H4/IC31 [35,36].The H4:IC31 vaccine induced antigen-specific CD4⁺ T cell and cytokine production that persisted 18 weeks after the most recent vaccination [37].The most prominent CD4⁺ T cell growth, IFN-γ production, and multifunctional CD4⁺ Th1 response has been shown after two doses of H4:IC31 containing 5, 15, or 50 g of H4 in addition to the 500 nmol IC31 adjuvant dosage [37].

The H4-IC31 vaccine is thought to enhance memory responses brought on by the BCG immunization in infants. Following stimulation with H4-IC31, peripheral blood mononuclear cells from BCG-immunized people were chosen for use in the cytokine secretion assay and their responses were assessed by monitoring the release of IL-17a, IFN-γ, IL-2, and TNF-α. The majority of BCG-vaccinated donor-derived peripheral blood mononuclear cells responded to H4-IC31 with a detectable IFN-γ response, with at least somewhat increased response in the presence of adjuvant IC31® and decreased response to corrupted vaccine [38].

### 2.4 Hybrid 56 + IC31

A protein-adjuvant vaccination known as H56/IC31 is combined of H56, a hybrid protein including the latency-associated proteins Rv2660c, Ag85B and ESAT6. Hybrid 56 + IC31, a protein-adjuvanted vaccine, combines the adjuvant IC31 with H56, a fusion protein made up of Rv2660c, Ag85B, and ESAT6 [39].

An Ag85B/Rv2620/ESAT-6 recombinant protein vaccine called H56 adjuvanted with IC31 (IC31+ H56) was found more efficient at triggering Th-1 cell responses in latently infected individuals than the higher dose (50g IC31+ H56). The efficiency of lowest dose (15g H56 + IC31) of the two doses has been shown in QuantiFERON-positive (QTF+) people [40]. Vaccination of healthy or infected adults with MTB with two or three doses of IC31+ H56 vaccine has produced durable stimulation of CD4⁺T cells and efficient, acceptable and effective immunity [41]. Based on the phase 1b study conducted in TB-free adults, the immunity induced by H56:IC31 and H4:IC31 vaccines and repeated with the BCG vaccine, in terms of efficacy and safety, it was observed that the H56:IC31 and H4:IC31 vaccines induced CD4⁺ T cells identifying vaccine-matched antigens and H56 and H4 specific IgG binding antibodies. In the H56:IC31 and H4:IC31 vaccination groups, CD4⁺ T-cells that recognized Ag85B had the highest frequencies of vaccine-induced responses. Vaccination with H56 and H4 and repeated with BCG produced a strong and multifunctional response against BCG specific antigens without change in the level of specific IgG against H56 and H4 [42].

### 2.5 ID93 + GLASE

ID93/GLASE is a vaccine that include protein and adjuvant. ID93 made a hybrid-protein containing Rv3620, Rv2608, Rv1813 and Rv3619, intermixed with the glucopyranosyl-lipid adjuvant-lasting stable emulsion (GLASE) [43]. The ID83 and ID93 chimeric fusion-proteins have been found as antigens for TB-vaccine and induced the IFN-γ response from the leprosy patients and from healthy in-house contacted of multibacillary-patients (HHC), while not from unexposed healthy-controls. Vaccination in mice with a protein-mixed with a Toll-like receptor-4 ligand (TLR-4L) comprising adjuvant GLASE motivated IFN-γ releae from Th1-cells. By experimental infecting the immunized mice with *Mycobacterium leprae*, both cellular penetration in the local lymph-nodes and in-place growing bacterial growth at the site were decreased in comparison mice that have not been immunized. Therefore, utilization of ID93/GLASE and ID83/GLASE as TB designated vaccines may lead to a cross-protective effect on leprosy [44]. The candidate vaccine of ID93/GLASE TB has been examined for the MTB clinical-strain K (MTB-K) from the family of Beijing (the most common strain of MTB in South Korea) that showed the ability of ID93/GLASE to stimulate constant Th1-biased immune pathway of antigen-specific multifunctional CD4⁺ T-cell co-generating TNF-α, IFN-γ and IL-2, especially IFN-γ, which exhibited a significant response up to 10 weeks after exposure [45]. Furthermore, ID93/GLASE usage as a BCG-prime-promoting regimen has been shown to be induced a remarkable sustained protective response against the hyper-virulent Beijing TB [46]. MTB HN878, a virulent strain from the family of W-Beijing, has been evaluated to identify its post-infection virulency and host immune-responses stimulation in animal models. MTB HN878 leaded to death or intense lung pathogenicity in C57BL/6-infected mice, while the lab-adjusted strains, (for instance, H37Rv MTB) do not. ID93/GLASE (as Th1 CD4⁺T cells generative) has been indicated for inducing the immunity for long-term that inhibits both early and late (4-week and 8-month, respectively) follow-up HN878 MTB pulmonary pathogeny [47]. Following low-dose HN878 aerosol infection, 80% of ID93/GLASE-vaccinated animals survived longer and had lower bacterial loads in their lungs [47]. A clinical trial phase-1 (random, double-blind, and dose-increasing) for the evaluation of ID93 antigen two-dose levels was implemented intramuscularly alone or combined with GLASE adjuvant two dose levels in 60 BCG-naive, and QuantiFERON-negative as well as uninfected adults in the US (ClinicalTrials.gov identifier: NCT01599897). All treatments induced both cellular and humoral vaccine-specific immunity [48]. In comparison with ID93 alone, ID93/GLASE enhanced the higher levels of ID93-specific antibodies, IgG1 and IgG3. Further GLASE supplementation also increased the frequency and multifunctional cytokine profile of CD4⁺ T-cells. GLASE adjuvant has been shown to induce functional Th-1 type cellular and humoral responses a desirable safety profile [49] (Table 1).

**Table 1:** Characteristics of tuberculosis vaccines containing protein and adjuvant.

Candidate	Vaccine composition	Immune response	Clinical trial phases	Goal	Route of administration
M72 + AS0	Mtb39a-Mtb32a fusion protein + AS01E adjuvant	CD4⁺ T cell, CD4⁺ and CD8⁺ T cell,	Phase 2b	Boost, post infection	Intramuscular
Hybrid + IC31	ESAT6-Ag85B fusion protein + IC31 adjuvant	TH1 (IFN-γ, IL-2)	Phase 2b	Prime, boost, post infection	Intramuscular
Hybrid 4 + IC31	Recombinant fusion of Ag85B-TB10.4 in IC31 adjuvant	CD4⁺ T cell (IFN-γ, TNF-α and IL-2)	Phase 2a	Boost	Intramuscular
Hybrid 56 + IC31	Ag85B-ESAT6-Rv2660c fusion protein + IC31 adjuvant	CD4⁺ T cell	Phase 2b	Prime, boost, post infection	Intramuscular
ID93 + GLASE	Rv2608-Rv3619-Rv3620-Rv1813 fusion protein + GLA-SE adjuvant	TH1 (IFN-γ, TNF-α and IL-2) CD4⁺ T cell	Phase 2a	Boost, post infection, therapeutic	Intramuscular

### 2.6 2.6.H1/CAF01

At present, H1 is undergoing phase II clinical trials in combination with the IC31 adjuvant. Additionally, it is in phase I clinical trials with the CAF01 adjuvant [50]. CAF01 is an adjuvant that promotes Th1/Th17 responses [51]. CAF01 (formerly known as DDA-TDB or Lipovac) is a promising adjuvant. CAF01 consists of cationic liposomes formed by the quaternary ammonium lipid N, N′-dimethyl-N, N′- ioctadecylammonium (DDA), incorporating the synthetic mycobacterial immunomodulator α, α′-trehalose 6,6′-dibeheneate (TDB). In comparison to a range of commercially available adjuvants, CAF01 has proven particularly effective in eliciting robust Th-1 and Th-17 T cell responses, along with effective humoral responses [52]. In preclinical trials of the H1:CAF01 vaccine, the CAF01 adjuvant exhibited the generation of substantial parenchymal IFN-γ producing T-cells. It also induced Th17-dependent memory and conferred protection when the vaccine was administered either before or after exposure to MTB. In human trials, H1:CAF01 elicited multifunctional antigen-specific T-cell responses that persisted for up to three years post-vaccination [53]. In a study, a solitary boost of BCG with the CAF01-adjuvanted fusion protein Ag85B-ESAT6 (H1) has been reported to be induced a long-term protection in comparison to BCG alone that may be linked to a specific induction, expansion, and sustenance of CD4 KLRG1− central memory cells that secrete IL-2 [54]. A phase I clinical study has been conducted to assess H1:CAF01 in healthy adult aged between 18 and 55 years who had not received the BCG vaccination [55]. The vaccination combining CAF01 with the H1 fusion protein has been reported to be induced the development of long-lasting T-cell immunity, primarily characterized by IL-2 and TNF-α producing T-cells [56].

## 3 TB candidate vaccine with mycobacterial whole cell or extract

### 3.1 M. Vaccae

*Mycobacterium vaccae* (MV) belongs to MTB genus found in soil usually as a nonpathogenic species. It shares several antigens with MTB and non-TB mycobacteria. The effectiveness of injectable and oral *M. vaccae* products have examined in various evaluations for the treatment of leprosy, TB and other disease such as depression and cancer [57]. As part of a clinical trial's third stage, it has been shown that *M. vaccae* induces humoral responses but variable INF-γ due to the HIV viral load, the number of CD4 T cells and any prior TB therapy [39]. MTB can be eliminated and inhibited by MV through improving Th1/Th2, activating the immune response mediated by the cytokine Th1, and activating MTB macrophage phagocytosis. In terms of pharmacoeconomics, even though the cost of treating MDR-TB with MV and chemotherapy increased, the incremental cost was lower because the curative outcome was considerably better [58]. A serious side effects have not been reported for MV and it is well tolerated. Also, studies have been shown that MV is safe and immunogenic in HIV-positive adults and induced CD4⁺ T-cell-expressing IL-10 and IFN-γ responses in the culture of MV-treated mice compared with those without MV [59,60]. Regarding the efficacy of MV, it should be highlighted that it can raise the rate of sputum smear conversion in patients with pulmonary TB in 1 or 2 months and 6 months following treatment [61]. Due to the fact that the majority of studies have emphasized the suppression of *mycobacterium* and host immune system interventions have been somewhat neglected, TB-based immunotherapy treatments are more needed. TB vaccine in tableted form (V7) as oral pill containing MV (NCTC11659) which has been killed by heat, has been shown promises to improve and shorten routine TB treatment regimens by create safe and fast-acting immune supplement in clinical studies [62].

### 3.2 DAR-901

DAR 901 is *Mycobacterium obuense*, inactivated by heat. In order to assess the DAR 901 safety, immunogenicity, and tolerance to numerous vaccination treatments at various dosage levels, ranging from 0.1 to 1 mg, a stage I trial was started in 77 adults who had previously received BCG and were HIV positive or HIV negative (ClinicalTrials.gov identifier: NCT02063555) [5]. it was reported that three doses of DAR 901 as a BCG booster have an appropriate safety profile and are well tolerated. Additionally, DAR-901 stimulates cellular and humoral immunity to poly-antigenic mycobacterial lysates [63]. In mice, DAR-901 induced IFN- and antibody responses. Defects in IFN- expression has been indicated to be associated with increased vulnerability to mycobacterial infection, and IFN- responses against several mycobacterial antigens predict protection from HIV-associated TB, even if the immunological correlates of vaccination protection against TB are unknown [64]. Immunization with DAR-901 improved antibody responses to DAR-901 but not to lysate or pure protein derivative of MTB. DAR-901 at 1 mg boost offered more defense against aerosol challenge in mice primed with BCG than homologous BCG boost [64]. DAR-901 recipients demonstrated an increase in polyfunctional or bifunctional responses by T cells against the DAR-901 antigen in comparison to the baseline. Higher levels of vaccine-specific CD4⁺ IFN, IL2, TNF-, and any other cytokine have been reported to be induced at seven days after the third dosage, . Th1 response predominated, with most responder cells having a polyfunctional effector memory pattern. While the more subdued DAR-901 response did not vary from placebo, BCG elicited larger CD4⁺ T cell responses than the control. Both DAR-901 and BCG failed to significantly or persistently increase Th17/Th22 cytokine responses. These findings imply that inducing larger levels of CD4⁺ cytokine activation may not be a crucial or necessary attribute for potential booster doses of the TB vaccine [65].

### 3.3 RUTI

Vaccine therapy is designed in order to stop latent infection and decrease the use of chemotherapy agents. RUTI as a fragmented and detoxified MTB cells, is a poly-antigenic therapeutic vaccine which it delivered in liposomes [39]. RUTI was used in combination with isoniazid a shorter course of latent TB infection treatment [66]. Usually a powerful Th1 reaction is induced by a decrease in the regulation of the Th2 response following RUTI administration, with the induction of immunological memory [67]. When the RUTI used after chemotherapy in guinea pig and murine models, it can decrease bacillary load. Also, when patients without latent TB and healthy adults both received the RUTI, it is immunogenic. Moreover, RUTI in the spleen, stimulates increase production of IFN-γ secreting cells and specific CD4⁺ and CD8⁺ effector T cells. While, RUTI in the lungs of infected mice, cause restricted dissemination of bacilli. RUTI generates a balanced immune response of Th1–Th2–Th3 instead of the pure Th1 response, which should be more efficient. The effectiveness of RUTI is better determined after chemotherapy. So that, during the course of chemotherapy, with a decrease in MTB load, In the lungs, there is a localized inflammatory response and a reduction in the immunological response., which is usually caused by the growth of bacilli. But when, RUTI use to after chemotherapy, increased the amount of CD8⁺, CD4⁺ and IFN-γ PPD-specific population, while in use of BCG after chemotherapy only CD4⁺ and IFN-γ PPD-specific population increased. Immune response of mice treated with RUTI and BCG has been shown BCG has no effect if administered therapeutically because RUTI produces a higher response against Ag85B, ESAT-6, and PPD [68]. Prabowo et al. exhibited RUTI vaccine's ability to prevent mycobacterial infection by balancing M1/M2 monocyte activity. The RUTI vaccination cause increase of mRNA expressions of Ly6C--related transcripts (Itgax, Nr4a1, Bcl 2, Pparg) and non-classical Ly6C- monocytes in the spleen [69].

### 3.4 MIP/MW

Heat-killed *Mycobacterium indicus pranii* vaccine (MIP) is used as an addition to conventional multidrug therapy and to prevent the spread of leprosy among close contacts of leprosy patients [70]. In India, a phase III safety and efficacy trial for the protection of pulmonary TB in healthy family members of TB patients with positive sputum swabs is now being conducted [71]. It has been shown that the MW vaccine in combination with chemotherapy increases pro-inflammatory cytokines such as IL-12, IFN-γ, TNF-α and IL-2 and decreases the effect of anti-inflammatory cytokines like TGF-β and IL-10 and can shorten the course of MTB treatment [72]. In macrophages infected with MTB using the MIP vaccine increases the induction TLR-4 down-stream signaling and facilitates P38 MAP kinase activation [73]. Saqib et al. investigated the effects of the MIP vaccine as a booster of the BCG in animal model and observed that pro-inflammatory cytokines such as IL-12, IFN-γ and IL-17 showed a higher increase in the group that received the MIP vaccine and BCG compared to other groups. It is induced more effective and stronger immunity against MTB [74]. Also, in another study, it was shown that encapsulating BCG and MIP inside alginate and exposing mice to the aerosol of this compound creates a strong and effective immunity [75] (Table 2).

**Table 2:** Whole-cell or extract tuberculosis vaccines.

Candidate	Vaccine composition	Immune response	Clinical trial phases	Goal	Route of administration
M. vaccae	Whole-cell *Mycobacterium vaccae*	IFN-γ and IL-10	Phase 3	Therapeutic	Intramuscular
DAR-901	Mycobacterial–whole cell or extract	IFN-γ, CD4⁺	Phase 2b	Boost	Intradermal
RUTI	Fragmented *Mycobacterium tuberculosis*	TH1 (IFN-γ), TH2, TH3, CD4⁺ and CD8⁺ T cells	Phase 2a	Boost, post infection, Therapeutic	Subcutaneous
MIP/MW	Inactivated *Mycobacterium indicus pranii*	IL-12, IFN-γ, TNF-α, and IL-2	Phase 3	Therapeutic	Subcutaneous/aerosol

## 4 TB candidate vaccine with attenuated *Mycobacterium Tuberculosis*

### 4.1 MTBVAC

In January 2013, the first vaccine with the attenuated human MTB bacillus known as MTBVAC entered in human clinical trials [76]. There has two independent stable genetic deletions for attenuation of MTBVAC, lacking antibiotic resistance indicators, that include the genes *phoP* and the genes f*adD2*6 that encoded the transcription factor PhoP and the cell-wall lipids phthiocerol dimycocerosates (PDIM), respectively [77].

Human monocytes are stimulated by MTBVAC to secrete pro-inflammatory cytokines such IL-6, IL-1, and TNF-α in a dose-dependent manner. It is also demonstrated that the use of a high dose of MTBVAC caused stimulation of the cells to the production of low amounts of IL-10. Gentamicin, an antibiotic that can impact mycobacteria's viability, has no impact on the stimulation of cytokine production in the MTBVAC culture medium., as a live attenuated strain of MTB Raquel [78]. It was shown that the absence of gentamicin in the cell culture medium with the exception of decreased IL-1β concentration, which is most likely related to extracellular mycobacteria, had no significant effect on cytokine production after acute stimulation. Compared these effects with BCG Pasteur, they found that the secretion of IL-6, IL-1β, IL-10, and TNFα is induced by MTBVAC. Therefore, vaccination with MTBVAC can improve the nonspecific protective effects of BCG vaccination by generating specific strains against MTB infection and exerting metabolic and epigenetic effects on the immune system [79]. Based on the available results, if MTBVAC is better protected against TB, it could be a reliable candidate for mass vaccination as a possible alternative to existing BCG vaccines [80] (Table 3).

**Table 3:** Features of live attenuated TB vaccines.

Candidate	Vaccine composition	Immune response	Clinical trial phases	Goal	Route of administration
MTBVAC	Live-attenuated *Mycobacterium Tuberculosis*	IFN-γ, IL-1β, IL-10, IL-6 and TNFα	Phase 2a	Prime, boost, post infection	Intranasal and mucosal

## 5. viral vectored TB candidate vaccine

5

### 5.1 TB/FLU-04l

Recombinant vaccine TB/FLU-04L comprises Ag85A/ESAT6 antigens released by MTB but not BCG, as well as the replication-deficient influenza virus strain A/Puetro Rico/8/34H1N1. Following the success of the phase I study, phase IIa clinical studies are now being conducted [81,82]. A phase 1 experiment using a TB vaccine based on an influenza vector that expresses Ag85A and ESAT-6 (TB/FLU-04L) has been revealed that the attenuated influenza virus can be a promising candidate to use as a vaccine vector due to its favorable safety profile in humans and high immunogenicity. This is achieved by truncating the viral NS1 protein. A replication-deficient, genetically stable influenza viral vector carrying ESAT6-Ag85A has been fabricated, which enhanced antigen-specific IFN- responses in mice and cynomolgus monkeys. In a clinical phase 1 study, the nasal spray vaccination was examined, and it was discovered to be well-tolerated with no significant side effects or virus shedding noted. Localized nasal cytokine production (IL-1, TNFα, and IL-2) was identified as an early sign of vaccination-induced immunity. After two vaccinations, memory CD4 and CD8 T-cell responses specific for the antigen were seen. While T-cell responses to Ag85A peaked at 21 days and then started to decline, those to ESAT-6 peaked at 21 days but continued to be present for the duration of the trial. There was no apparent antibody response to the influenza vector [[82], [83], [84]].

### 5.2 MVA85A

A recombinant strain of the Vaccinia virus Ankara that can express the MTB antigen 85A is referred to as a viral vector by the name MVA85A. Phase I studies of this vaccine in adults with HIV infection in Senegal revealed a well-tolerated and immunogenic effects but, a few participants have reported experiencing mild local and systemic side effects [5]. MVA85A has been applied as a heterologous boost for BCG vaccine. Boosting BCG with MVA85A enhanced BCG-induced protection against TB infection in animals. Phase IIb studies in 2797 infants showed MVA85A was well-tolerated and immunogenic with a poor protection against TB infection has been found [85]. In phase 2 trial in 650 infected adults with HIV-1, MVA85A has been reported safe, well-tolerated and immunogenic. However, did not have any suitable impact against TB disease or MTB infection [86]. MVA85A was able to induce a polyfunctional CD4⁺ T-cells population, expressing INF-γ, IL-2, TNF-α), IL-17 and granulocyte-macrophage colony-stimulating factor and a modest CD8⁺ T-cells response [87]. Aerosol vaccination with MVA85A is have several advantages like feasibility, safety, and can cause systemic cellular immune responses that are specific to mycobacteria in mucosa [88]. Wajja et al. [89] utilized MVA85A among adult participant with prior BCG vaccination, and with and without Schistosoma mansoni (SM) infection. With no difference in cellular immunogenicity between adult participants with and without an active SM infection, MVA85A elicited a potent cellular response. The results indicated that SM infection did not inhibit cellular immunogenicity of MVA85A. This is important given the poor efficacy of BCG in tropical latitudes. Furthermore, MVA85A was safe in this population of African adolescents [90]. In a prior work, aerosolizing MVA85A has been reported to be more effective than intradermal vaccination at eliciting comparable systemic Ag85A-specific T cell responses and higher frequencies of mucosal Ag85A-specific T cell responses. Heterologous vaccination, however, did not seem to decrease these effects (either pre- or post-intradermal vaccination) [91]. These findings imply either that BCG cannot further boost Ag85A-specific T-cell responses that have already been maximally primed by MVA85A or, alternatively, that MVA85A is more immunogenic when given as a boost vaccination after BCG priming. The control and MVA85A groups both experienced similar levels of Ag85A-specific T-cell responses after receiving the BCG vaccine. According to Nemes et al. [92] MVA is a good vector for a given antigen just like BCG, but the immune response to Ag85A in this newborn cohort is subpar. It is significant to note that MVA85A prime had no negative effects on the immunogenicity of BCG in terms of the quantity, functionality, memory profile, and proliferative ability of antigen-specific CD4⁺ T cells.

### 5.3 Crucell 35 CE+ MVA85A

Crucell 35 CE/AERAS-402 isa replication-deficient adenovirus 35 contained a fusion protein of the mycobacterial antigens 85A, 85B, and TB10.4 [93]. This TB vaccine was shown predominantly to induce CD4⁺ T-cells expressing three (IFN-γ, TNF-α, IL-2) or two (IL-2 and TNF-α) cytokines. AERAS-402/AD35.TB-S induced potent antibody responses specific to Ag85A and Ag85B, especially following the second doses [94]. AERAS-402/AD35.TB-S mostly produced polyfunctional CD4⁺ and CD8⁺ T-cell responses with adequate safety and durability [95]. As of now, Crucell 35 CE/AERAS-402 is undergoing a phase II trial involving HIV-infected, BCG-vaccinated adults in South Africa [96]. While AERAS-402 is largely immunogenic, Nyendak et al. [97] emphasized that the amount to which these cells are capable of detecting the MTB-infected cell may not be accurately reflected by their polyfunctionality. One of the fundamental investigations on how a potential MTB vaccine protects individuals with active or prior pulmonary TB, demonstrated that people receiving therapy for active pulmonary TB or those who have just finished treatment can safely get the AERAS-402. There were no knock-on effects, particularly severe ones similar to the Koch phenomenon [98].

### 5.4 Ad5 Ag85A

Ad5Ag85A as a recombinant strain of replication-deficient adenoviral vector expresses the MTB Ag85A. Intranasal delivery of Ad5Ag85a has been shown to be protected mice against pulmonary TB infection effectively either as a booster vaccination for BCG or as a primer. Intranasal Ad5Ag85A vaccine has been reported to be induced a higher effective immunity response against TB than intramuscular or subcutaneous vaccination with Ad5 expressing Ag85A (5-8 CE5A) [99,100]. Adam et al. [101], revealed that intradermal and endobronchial administration of 5-8 CE5A induced similar peripheral blood antigen-specific responses. BCG vaccinated cattle boosted with 5-8 CE5A had no significant differences in Ag85A-specific CD4⁺ T cell lines compared to those acquired before to boosting in terms of cytokine production that promotes inflammation, cytotoxic compounds, or mycobacterial control. Yet, Ag85A-specific CD4⁺ T cell lines of post-boost produced majority of the immune regulatory cytokine IL-10 than CD4⁺ T cell lines prior to viral boosting. Accordingly, the protection acquired through restricting mycobacterial growth by increased number of Ag85A-specific CD4⁺ T cells may stem from anti-inflammatory properties to restrict immune-pathology [102].

Phase 1 trial studied the immunogenicity and safety of AdHu5Ag85A in both BCG-naïve and BCG-immunized healthy adults. Safety and tolerance of the AdHu5Ag85A intramuscular immunization was confirmed in both trial volunteer groups. Additionally, although AdHu5Ag85A was immunogenic in both trial volunteer groups, a much more boosted immunity via poly functional CD4⁺ and CD8⁺ T cell has been shown in previously BCG vaccinated group. Moreover, in contrast with preexisting anti-AdHu5 humoral immunity, some evidence had been shown that such preexisting immunity significantly reduced the potency of AdHu5Ag85A vaccine [103].Mu et al. [16], demonstrated that the fusion protein that AdAg85A-TB10.4 encodes was effectively produced and that it elicited T-cell reactions particular to particular antigens. What's more, TB10.4 immunization was demonstrated to elicit a level of immunological protection that was much greater than that by AdAg85A or traditional BCG immunization. This is due to the activation of both Ag85a- and TB10.4-specific T cells by AdAg85A [16].

### 5.5 ChAdOx1 85A

Replication-deficient adenovirus-originated vectors from chimpanzees are considered as favorable modern kind of genetic-carrier vaccines. Adenovirus-originated vectors are a strong choice for delivery of the vaccine of TB as the natural respiratory-tract viruses that can aim at the lung, the basic site of MTB colonies. Adenovirus with innate adjuvant property is able to simultaneously express different antigens and have an adoptable secure margin. Besides, both intense humoral and cell-mediated responses can be resulted from these viruses. However, the clinical application of human-targeted adenovirus for vaccine matrix is limited due to the primary immunity of lots of populations that reduces the effectiveness of such a vaccine [104]. Discontinuing of the clinical trial (STEP-trial) of the adenovirus-based (type 5, Ad5) vaccine HIV-1 in 2007 due to both the absence of positive affect and higher HIV-infection in vaccinated individuals with previous Ad5 immunity and/or circumcised individuals has lowered the interest in the development of Ad5-based vaccine. ChAds have been considered as alternatives since humans show none/low degrees of neutralizing antibodies. ChAds, despite being of a distinct species, have a respiratory tract tropism that allows them to infect people. ChAd-vectors have been reached the clinical application level and have presented the capability of stimulating the immune responses against encoded antigens, proper safety, and easy production at a large-scale [104]. The ChAdOx1 vector by expressing NP and M1 antigens of influenza has been recognized for its safety and immunogenicity in a small phase-I (dose promotion trial). In vivo preclinically-studied murine have indicated consistent protection of ChAdOx1 85A as applied in the immunization regime of BCG-ChAdOx1 85A-MVA85A. The clinical studies of Phase-I are now evaluating the vaccination safety of ChAdOx1 85A alongside MVA85A infusion in BCG-vaccinated adults in the UK. The ChAdOx1 85A single-dose stimulates the ChAdOx1-specific IFN-y response without boosting by homologous vaccination. Vaccinating by ChAdOx1 85A led to remarkable Ag85A-specific IFN-γ, TNF-α, and CD8⁺ T cells, that MVA85A promoted them [105] (Table-4).

**Table 4:** Viral vectored tuberculosis vaccines and their characteristics.

Candidate	Vaccine composition	Immune response	Clinical trial phases	Goal	Route of administration
TB/FLU-04l	Influenza virus strain A/Puetro Rico/8/34H1N1 and MTb antigens Ag85A and ESAT6	Type 1 IFN-γ	Phase 2a	Boost	Intranasal and sublingual aerosol
MVA85A	Recombinant Vaccinia virus Ankara expressing Ag85A	IFN-γ, CD4⁺ T cell, CD4⁺ T cell (IFN-γ and IL-2), CD4⁺ T cell (IFN-γ, TNF-α, IL-2 and, IL-17)	Phase 1,2	Boost, post infection, therapeutic	Intradermal, Phase 2 Aerosol administration trials ongoing
Crucell 35 CE+ MVA85A	Modified adenovirus 35 expressing Ag85A-Ag85B-TB10.4 fusion protein	IFN-γ, CD4⁺ T cell	Phase 2a	Boost	–
Ad5 Ag85A	Recombinant adenovirus 5 expressing Ag85A	IFN-γ, CD4⁺ and CD8⁺ T cells	Phase 1	Prime, boost, post infection	Aerosol
ChAdOx1 85A	Chimpanzee adenoviral and Ag85A	IFN-γ, TNF-α, and CD8⁺ T cells	Phase 1	Prime, boost	Aerosol/Intramuscular

## 6 6.recombinant live TB candidate vaccine

### 6.1 VPM1002

A recombinant strain of BCG that produces membrane-penetrating listeriolysin is known as VPM1002 vaccine. This listeriolysin belongs to the bacterium Listeria monocytogenes which lacks the urease C gene and has hygromycin resistance marker. Compared with the parental BCG (pBCG), it has been shown to provide better protection against TB by stimulating type 1 and type 17 cytokines in mice [5]. Because the rBCG vaccine candidate VPM1002 can penetrate phagosome membranes by secreting Hly, it allows Mycobacterial antigens to enter the cytosol of the host cell and improve the antigen presentation. In the first step, a BCG expressing Hly was constructed. However, in this recombinant mutant, the biological activity of Hly is not ideal. Hly is only active at acidic pH. In order to counteract the neutralizing effect of BCG on phagosomes, the gene encoding urease C was omitted. In fact, the resulting rBCG-ureC-Hly mutant prepared the optimal pH for Hly in the phagosome. Mycobacterial antigens can enter the cytosol through phagosome membrane perforation and be processed through the major histocompatibility complex route for CD8 T-cell priming [106]. The cytokine-producing cell profiles have been reported be alike in the BCG and VPM1002 groups. The type of cytokine production (for IFN-γ, IL-2, and TNF-α) by CD4⁺ and CD8⁺ T cells has been described be consistent with the findings of phase I studies [107]. Phase II study confirmed safety and immunogenicity of VPM1002 in South African neonates unexposed to HIV. A dose of VPM1002 vaccine stimulated poly functional CD4⁺ and CD8⁺ T-cell profiles similar to BCG. Interestingly, the ratio of CD8⁺ IL-17 T cells were enhanced at 6 months after vaccination in specific group [108]. In a Phase II clinical trial, VPM1002-ZA-2.12TB, healthy, not exposed to HIV were randomized to receive VPM1002 or BCG vaccine. VPM1002 was safe and well-tolerated compared with BCG. Preliminary data analysis indicated that VPM1002 was at least as safe as, and reported be better tolerated than a single dose of BCG in newborn infants. VPM1002 also produced a strong T-cell response skewed towards Th1-type immunity like to what had been report in the previous Phase I studies [106]. In another study, VPM1002 in immunocompromised mice discloses a higher safety profile compared to BCG. Based on the findings of Phase II in newborns and Phase I trial in adults, VPM1002 is probably to be a stronger vaccine than BCG [109]. VPM1002 has been used in goats by the Friedrich Loeffler Institute in Germany to vaccinate them against *M. caprae* infection. Infections with *M. bovis* in cattle and *M. capraein* goats, are very important in animal husbandry and can also infect humans [110] (Table 5).

**Table 5:** Characteristics of recombinant live tuberculosis vaccines.

Candidate	Vaccine composition	Immune response	Clinical trial phases	Goal	Route of administration
VPM1002	Recombinant BCG strain	CD4⁺ and CD8⁺ T cell, IL-17	Phase 3	Prime, boost	Intradermal

## 7 Delivery systems in TB vaccination

Advanced or next-generation vaccines include the recombinant fusion proteins or DNA along with adjuvant added, to boost the immunogenicity against potential infectious diseases. For an efficient immune response, the successful vaccine delivery systems are essential which stimulate the uptake of vaccines quickly. Some of the advanced delivery systems have been discussed as follows. Appropriate vaccine administration/delivery is the key element to ensure successful vaccination. Some efficient delivery systems in TB vaccine includes: nanoparticle, liposome, virus-like particles (VLPs), virosomes, immune stimulating complexes (ISCOMs) and dendrimer-based have been introduced to accelerate vaccine uptake and more effectively stimulate the immune system.

### 7.1 Nanoparticles

Delivery systems based on nanoparticles increase the effectiveness of vaccines and stimulate the immune system by protecting antigens and forming antigenic storage. The potential of nanoparticles to modulate immune responses to attain appropriate outcome is important feature for their usage in vaccines formulation. To increase and improve protective immune responses, nanoparticles may be used as a delivery approach or as an immune-inducing adjuvant. Some nanoparticles have been shown to stimulate antigens uptake and present by APCs keep the antigens morphology from enzymatic cleavage. Antigens-loaded nanoparticles may also possess a local store role, extending the length that the antigen is presented to immune cells [111,112].

#### 7.1.1 Chitosan

Chitosan-based, polymeric/polyester-based and cationic nanoparticle are examples of these systems. Mannosylated chitosan (MCS) nanoparticles cause strong stimulation of cellular immune responses and increase the secretion of cytokines such as IFN-γ, TNF-α and IL-2 [113]. Chitosan nanoparticles containing early secretory antigen target (ESAT-6) indicated ability to promote higher levels of IFN-γ, IL-4, and IgG compare to control [114]. Chitosan nanoparticles have been shown efficiency in pulmonary delivery of DNA plasmid encoding eight HLA-A*0201-restricted T-cell epitopes of MTB by inducing DCs maturation due to release of DNA from chitosan nanoparticles. Pulmonary administration of this system has shown significantly higher induction of IFN-γ production than pulmonary administration of plasmid as well as intramuscular administration route [115].The administration of MTB lipid-bound chitosan nanoparticles in mice has shown to induce cytokines secretion of prominent Th-1 and Th-2 cells in lymph node and spleen, and also significantly increased levels of IgG, IgG1, IgG2 and IgM than control animal. Moreover, significantly increased γδ-T-cell response has been reported in mice lymph node cells after immunization by MTB lipid-bound chitosan nanoparticles than controls inoculated with free chitosan nanoparticles or MTB lipids [116].

### 7.2 2 Polylactic-co- glycolic acid (PLGA) and polyethyleneimine (PEI)

Polylactic-co- glycolic acid (PLGA) is a degradable polymer that is made of smaller monomeric units, and due to its flexibility, biodegradability and small size, it is an attractive option for encapsulating multiple antigens and preventing their degradation in the human body environment. For the delivery of drugs and vaccines in humans, PLGA have the approval of the US Food and Drug Administration [117,118]. PLGA–polyethyleneimine (PEI) has been used for pulmonary delivery DNA vaccine encoding the Rv1733c that is an MTB latency antigen. This system indicated the ability to increase of human DCs maturation and induce the release of IL-12 and TNF-α by DCs comparable to levels detected following lipopolysaccharide (LPS) induction. It has also enhanced lungs T cell proliferation and IFN-γ release in animal model in response to Rv1733c DNA adsorbed to PLGA–PEI. The highest immunogenicity has been reported using pulmonary priming with PLGA–PEI adsorbed Rv1733c DNA after boosting with Rv1733c protein [119]. PLGA and dimethyl dioctadecylammonium bromide (DDA) hybrid nanoparticles loaded with HspX/EsxS antigens with and without monophosphoryl lipid A (MPLA) has been evaluated to immunize mice against MTB. This system has shown effective activation of Th1, Th17 and increased IgA, IgG1 and IgG2a release both alone or as a booster for BCG [120]. Fe3O4-Glu-polyethyleneimine (PEI) nanoparticles have shown considerable potential to delivery of DNA vaccine of Ag85A and ESAT-6 MTB antigens and IL-21 immunoadjuvant. Fe_3_O_4_-Glu-PEI nanoparticles-based formulation has shown a statistically significant higher protective effects in the immunized mice against MTB than DNA vaccine Ag85A-ESAT-6-IL-2 [121].

### 7.3 3 Pluronic-stabilized poly (propylene sulfide)

The results of *in vitro* and *in vivo* studies have been shown improved activation of antigen-specific CD4⁺ and CD8⁺ T cell by Pluronic-stabilized poly (propylene sulfide) (PPS) NPs. PPS NPs conjugated the TB antigen Ag85B in combination with the oligonucleotide CpG adjuvant has been designed as a delivery system of vaccine. It has been reported that PPS NPs incorporation increased the efficiency of Ag85B vaccine in promotion of Th1 and Th17 responses and polyfunctional CD4⁺ T cell reaction, particularly for pulmonary administered. PPS NPs has been reported to have ability of inducing *in vitro* DCs maturation and significantly increased the adjuvant property of oligonucleotide CpG in a concentration-dependent mechanism [122].

### 7.4 4 Guar-gum nanoparticle

Guar gum is natural, biodegradable and a water-soluble polysaccharide with a high-level affinity to mannan receptors on DCs, Langerhans cells, monocytes and endothelial cells because of the presence mannose moiety. Guar gum has been broadly used for delivery of antigen via oral route because of its low toxicity and potential in encapsulation a high concentration of antigens. It is also cost-effective. Furthermore, excellent swelling property of guar gum at acidic condition provide an effectiveness to keep the loaded antigen from unforgiving gastrointestinal environment. Ag85A-incoperated guar gum nano-system has been developed for oral vaccination against TB that indicated the protection the Ag85A antigen from acidic degradation and merely 12% release of antigen after 4 h Ag85A-loaded guar gum nano-system has also shown a significantly improved *in vivo* immune response after oral administration than plain antigen and a significant increased level of IgA in the salivary, nasal and vaginal secretions after 42 days of primary administration [123].

### 7.5 5 Mesoporous Silica Nanoparticles (MSNs)

Mesoporous Silica Nanoparticles (MSNs) have been prepared and widely examined as nano-system of delivery of MTB vesicle-associated proteins. The covalent anchorage of the immunomodulatory proteins of Ag85B, LprG and LprA had been illustrated to the surface of the MSNs. The efficiency of MSNs as carrier of vesicle-associated proteins-based vaccine has been shown by determining of TNF-α and IL-10 levels in treated macrophages [124].

### 7.6 Liposome

Liposome is one of the suitable structures for drug and vaccine delivery due to its unique properties such as distinctive features is that the compound carries both hydrophobic and hydrophilic nature [125]. Liposome also protect antigens and prevent their destruction in the host body and increase their supply to APCs. Liposome has been used in the delivery of TB candidate vaccine containing antigen ESAT 6 and Ag85A along with IC31 adjuvant as well as RUTI vaccine and has revealed high efficiency and has shown a significant increase in both humoral immunity and cellular immunity [[126], [127], [128]]. The results of a clinical trial in humans have been shown that Ag85B-ESAT-6 adjuvanted with liposome adjuvant CAF01 promoted long lasting T-cell response featured by mostly IL-2 and TNF-α expressing T-cells [56]. The potential of 1, 2-dioleoyl 3-trimethylammonium ropane (DOTAP) as an adjuvant has broadly been investigated in vaccine-based studies. HspX, PPE44 and EsxV loaded DOTAP liposome has shown ability to significant induction of IL-17, IFN-γ and IL-12 levels and higher IgG2a/IgG1 ratios in BCG-primed mice [129].Cationic liposomes contained *mmaA4* gene deletion mutant of BCG (ΔmmaA4BCG) significantly increased the frequency of CD4 T cells expressing both IFNγ and TNFα or IFNγ, TNFα and IL-2 compared to CD4 T cells derived from animal model immunized with BCG [130]. DMT is an effective inducer of Th1-depended immune response consisted of dimethyldioctadecylammonium (DDA) and two pattern recognition receptor agonists monophosphoryl lipid A and trehalose 6,6′-dibehenate (TDB). DMT has been used a liposomal adjuvant to improve efficiency of DNA vaccine encoding Rv2875, Rv3044, Rv2073c, and Rv0577 antigens of MTB in C57BL/6 mice [131]. The liposomes containing HspX, with or without MPLA or CpG DNA adjuvants have been reported to have ability to induce the effective responses of humoral and cellular immune, principally by promoting THE expression OF INF-γ and TNF-α by both CD4⁺ and CD8⁺ cells. HspX and MPLA highly activated CD8⁺ cell and humoral reactions. L-HspX and L-HspX-CPG DNA -loaded liposomes have also been decreased both lung inflammation and the load of bacteria [132]. Liposomes containing DDA/TDB loaded with fusion protein (FP) of MTB has been shown the potential of increased levels of IFN-γ, IL-4, IL-17, and IL-12 in spleen tissue and increased levels of IgG2a, IgG1, and IgG2b in serum after three weeks following the last subcutaneous administration specially in the BCG-primed group [133].

### 7.7 Virus-like particles

Another delivery system in vaccines introduced for TB is Virus-like particles (VLPs). VLPs have received much attention in recent years in the topic of vaccination. And this attention is due to the unique characteristics that they have, for example, they do not change the structure of proteins, they activate both types of immune systems and they are able to imitate the structure of the virus from which they are made, as well as VLPs with the nanometer size is easily picked up by APCs and degraded and cause strong cellular immunity. So, VLPs are considered as an efficient and effective system in vaccine delivery [134,135]. VLPs show promising potential in vaccine advance due to their several features, together with but not restricted to their safety lacking the menace of infection, their capacity to mimic the size and structure of original viruses, and their capability to show antigens on their surface to improve the immunity. In a recent study, CFP-10 recombinant protein in combination with hepatitis B virus core protein-VLPs caused more and stronger stimulation of the immune system compared to the use of recombinant protein alone in Balb/c mice [136,137]. Hepatitis B virus core protein has been used to increase immunogenicity. Hepatitis B virus core- ESAT-6 has been shown to induce significantly higher levels of ESAT-6-specific antibodies and CD4⁺/CD8⁺ T cell immunity then ESAT-6 protein in animal models [138]. Human papillomavirus (HPV)16 L1 capsid protein has been studied to develop of a vaccine using the extracellular domain of Ag85B of MTB. The HPV16L1/Ag85B VLPs have been reported to be effective to induce the humoral and cellular immune response against MTB in in female C57BL/c mice [139]. Influenza A VLPs have been developed using a 20 amino acid sequence known to comprise a potent T-cell epitope from ESAT-6 of MTB, which induced the high level of serum antibody against ESAT-6 [140]. LV20 VLPs has been designed by inserting the fusion protein consisted from Heparin-binding hemagglutinin (HBHA) and MTP pili (MTP) into the receptor-binding hemagglutinin (HA) portion of influenza virus. LV20 VLPs in combination with the DDA and Poly I: C (DP) adjuvant have been induced significantly higher levels of antigen-specific humoral immunity and CD4⁺/CD8⁺ T cell responses in and decreased the MTB load in the lungs of animal models [141]. Parainfluenza virus 5 (PIV5) expressing MTB Ag85A and Ag85B have been prepared and studied to determine their immunogenicity and protective properties in an animal model. PIV5– Ag85A has been reported to decrease MTB load in lungs of animal models after single dose administration than bacterial load in unvaccinated and BCG vaccinated animals [142].

### 7.8 Virosome

Virosomes are artificial virus like, non-replicating and vesicular particles consisted of viral envelopes. Virosomes can activate immune response due to increase APCs antigen uptake and inducing immune cells. They have broadly studied to usage in directly delivery of vaccine antigens to the host cells. Microbial antigens used as vaccine candidate may be captured inside the virosome lumen or can be artificial cross-linked to surface of virosome. Virosomes are able to interact with and penetrate host cells and transport the vaccine antigen into the MHC class I antigen processing pathway due to the presence viral envelope proteins. Virosome are usually used as a bearer for the delivery of drugs or vaccines. Drug molecules or vaccines are placed in their inner cavity. Because of the layer made of glycoprotein and phospholipid in the outer cover, they can easily interact with the cell and enter the target cells. Because of their non replicative nature, biodegradable, adjuvant, and noninfectious in the host they are a good choice for delivering vaccines. They induce humoral immune responses and CD8⁺ and CD4⁺ cells [143]. Plasmid pAAVCMV, which contains the gene encoding Ag85A antigen, was placed inside the Sendai virosome and was injected intramuscularly into mice, and after immunization, it was shown that CTL, Th1, and NK cells were well activated [144].

### 7.9 Immunostimulant complexes

Another substance that plays role as a carrier of antigens and adjuvant in vaccination is a lipid -based compound called immunostimulant complexes (ISCOMs). ISCOMs are advanced by combining the mixture of cholesterol, saponin and phospholipids in the suitable fraction [145]. These compounds can be used to deliver vaccines through the subcutaneous and mucous membranes to properly stimulate cellular and humoral immunity. Ag85B-loaded ISCOM containing Quil A (a saponin adjuvant) has shown significant ability to improve humoral and cellular immune responses against MTB infections in pulmonary immunized mice [146]. Use of CTA1-DD/ISCOM-associated antigen fusion protein Ag85B-ESAT-6 caused a strong induction of Th1 cells and the secretion of IFN-γ, which play an important role in protecting against TB infection [147]. ISCOMS containing the 38- kDa protein of MTB prepared with the high level of Quil A has shown ability to induce high levels of IFN-γ after activation of Th1cells in spleen cells of subcutaneously immunized mice [148]. HspX/EsxS of MTB along with ISCOMS nano-adjuvants and MPLA has been shown to enhance immunogenicity of MTB fusion protein after subcutaneous usage in animal model dur to induce more effective Th1 immune responses and the levels of IgG2a and IgG1 than MPLA adjuvant and also improved BCG effects as a BCG booster [149].

### 7.10 Dendrimers

Dendrimers are synthetic symmetrical macromolecules nanocarriers consisted of a core surrounded by the symmetric and homogeneous tree-like branches. Dendrimers have some functional groups located at the surface of the dendrimer that play an essential role high solubility and reactivity. Dendrimers also possess interior cavity that make them appropriate to use as delivery of biological molecules and drugs [150,151]. There are many different dendrimers, and each has different biological properties such as electrostatic interactions, self-assembling, polyvalency, solubility, low cytotoxicity, chemical stability, proper integration with phospholipid bilayers and the pharmacokinetics features including the drug internalization, bioavailability and their maintenance time make dendrimers a suitable choice in the health field [152,153]. It has been identified that dendrimers when combinates with adjuvant provide an active and long-lasting protection [154]. A polyamino acid called MAPs (multiple antigen peptides), which is used in the serological diagnosis of TB, was used as a dendrimer to carry ESAT-6 epitope and it was observed that it creates a strong immunity that can protect against TB [155].

## 8 Conclusion

Several of the introduced vaccines for TB have been reported to be able to reach the final phases of clinical trials, and these vaccines have succeeded in strongly stimulating the immune system and even caused long-lasting immunity in the host. The vaccines that go through the early phases of clinical trials have been able to create safe and efficient immunity that has been comparable to BCG, even better than that. With the introduction of new TB vaccines that are strong immune system stimulators and the use of efficient delivery systems, hopes for the treatment and prevention of TB are increasing. One of the ways to develop the effect of vaccines is to use efficient delivery systems, which have also been used in TB vaccines and have been able to play a significant role in increasing the effectiveness of TB vaccines. Nanoparticle, liposome, VLPs, virosomes, ISCOMs and dendrimer-based delivery systems have extensively been studied to accelerate vaccine uptake and more effectively induce the immune responses to MTB infection. Most of the studies on vaccine delivery systems have been performed in experimental models, so to confirm their efficiency in human host, there is a need for future surveys, especially on immunogenic properties and investigating their long-term side effects on the host.

## Funding statement

This study was supported by the Immunology Research Center (IRC), 10.13039/501100004366Tabriz University of Medical Sciences, Tabriz, Iran.

## Ethics approval and consent to participate

This study was approved by the Ethical Committee of Tabriz University of Medical Sciences, Tabriz (IR.TBZMED.VC.REC.1397.358).

## CRediT authorship contribution statement

**Rasoul Hoseinpour:** Writing – review & editing, Writing – original draft, Software, Methodology, Investigation. **Alka Hasani:** Writing – review & editing, Writing – original draft, Visualization, Software, Project administration, Methodology. **Behzad Baradaran:** Writing – review & editing, Methodology, Investigation. **Jalal Abdolalizadeh:** Writing – review & editing, Writing – original draft. **Roya Salehi:** Writing – review & editing, Software. **Akbar Hasani:** Writing – review & editing, Writing – original draft. **Edris Nabizadeh:** Writing – review & editing, Writing – original draft. **Mina Yekani:** Writing – review & editing, Writing – original draft, Investigation. **Roqaiyeh Hasani:** Writing – review & editing. **Hossein Samadi Kafil:** Writing – review & editing, Writing – original draft. **Khalil Azizian:** Writing – review & editing, Writing – original draft. **Mohammad Yousef Memar:** Writing – review & editing, Supervision, Software, Methodology, Conceptualization.

## Declaration of competing interest

The authors declare that they have no known competing financial interests or personal relationships that could have appeared to influence the work reported in this paper.
